# The physical activity paradox: a longitudinal study of the implications for burnout

**DOI:** 10.1007/s00420-021-01759-y

**Published:** 2021-10-06

**Authors:** Juriena D. de Vries, Arnold B. Bakker

**Affiliations:** Center of Excellence for Positive Organizational Psychology, Erasmus University Rotterdam, Woudestein Campus, Mandeville Building T16.17, PO Box 1738, 3000 DR Rotterdam, The Netherlands

**Keywords:** Burnout, Occupational stress, Work, Exercise, Physical job demands

## Abstract

**Purpose:**

This study investigates the independent and interactive associations of physical job demands and three types of off-job physical activity (during transportation, household, and recreation) with burnout. We use a recently proposed new conceptualization and assessment of burnout including core and secondary burnout symptoms. We predicted that physical job demands would be positively and the three types of off-job physical activity would be negatively related to burnout. Further, we hypothesized that the negative relations between the three types of off-job physical activity and burnout would be stronger for employees with low (vs. high) physical job demands.

**Methods:**

To test our hypotheses, we conducted a two-wave survey study among a heterogeneous sample of full-time workers (*N* = 355), using a longitudinal design with a half-year time lag. We tested cross-sectional, prospective and longitudinal relations.

**Results:**

Hierarchical regression analyses partly confirmed our predictions. Cross-sectionally and prospectively, it was shown that physical job demands were positively related to burnout symptoms. In addition, off-job physical activity was negatively related to primary and secondary burnout symptoms among employees with low physical job demands and positively related to burnout symptoms among employees with high physical job demands. However, these relationships disappeared when investigated longitudinally.

**Conclusion:**

Together, these findings suggest that not all off-job physical activities can prevent burnout, and that potential positive effects of physical activity during off-job time may depend on employees’ physical activity level at work.

**Supplementary Information:**

The online version contains supplementary material available at 10.1007/s00420-021-01759-y.

Burnout, a work-related phenomenon characterized by a severe loss of physical and mental energy (Leiter et al. 2014; Schaufeli et al. 2020), is prevalent and widespread (Eurofound 2018)—it can be found among many different occupational groups. Burnout may negatively affect employees and employers, in the form of employee depression (Toker and Biron 2012) and absenteeism (Ybesma et al. 2010). Early on, burnout was conceptualized as a syndrome of exhaustion, cynicism, and reduced professional efficacy (Maslach and Leiter 2016). However, the exact conceptualization and assessment of burnout is subject to debate (Bianchi et al. 2019; Kristensen et al. 2005; Schaufeli et al. 2009). Researchers recently proposed that burnout can best be conceptualized as the inability and unwillingness to spend effort at work. Accordingly, it is suggested that its core components are exhaustion, mental distancing, and cognitive and emotional impairment (Schaufeli et al. 2020).

In the present study, we sought to investigate predictors of burnout, using this new conceptualization and assessment of the syndrome. Poor working conditions, such as the combination of high job demands and low job resources, is a fertile ground for burnout (Bakker and Demerouti 2017). However, burnout may also arise if employees fail to use adaptive regulation strategies, such as recovery and job crafting, when dealing with their job demands (Bakker and de Vries 2021). Accordingly, it is essential to determine which regulation strategies may help employees deal with their ongoing job demands and prevent burnout.

We suggest that off-job physical activity may serve as such an individual self-regulation strategy. This idea is in line with research showing that physical activity has the potential to prevent and reduce burnout (Gerber et al. 2020; Naczenski et al. 2017; Ochentel et al. 2018). However, physical activity may not play the same role for all employees. When jobs are mainly characterized by physical job demands, employees will be physically active at work throughout the whole day and across the entire workweek. Increasing evidence indicates that physical activity at work impairs physical health, whereas off-job physical activity promotes it (i.e., the ‘physical activity paradox’; Coenen et al. 2020; Holtermann et al. 2018). These contrasting physical activity effects may apply to burnout as well, but, to date, these relations are not fully clear. Therefore, this study aims to investigate the relation between different domain-specific physical activities and burnout, which may help to understand how to use physical activity in an optimal way to prevent burnout. To this end, we conducted a survey study using a longitudinal design with a half-year in-between two waves of measurement. Full-time working employees answered questions about their physical activities on and off the job as well as experiences of burnout, which allows us to test temporal precedence (Spector 2019).

We aim to contribute to the literature in three ways. First, we aim to enhance insight into different domain-specific physical activities in relation to burnout. We focus on employees’ physical job demands as an indicator of physical activity at work. These job demands refer to physical aspects of the job that require sustained physical effort, which cost considerable energy and bear the risk of being unhealthy (Demerouti et al. 2001; Holtermann et al. 2018). Further, we focus on different off-job physical activity types (recreational, transportation, and household physical activity) in relation to burnout. We argue that—in general (i.e., for the average worker)—physical job demands are positively and off-job physical activities are negatively related to burnout. The reason for this is that physical job demands may require sustained physical effort without sufficient recovery time and therefore overtax employees’ energy reservoir (Holtermann et al. 2018; Sato et al. 2017; Xanthopoulou et al. 2007). In contrast, off-job physical activities may help gain resources that can be used to recover from and cope with job demands (Naczenski et al. 2017).

Second, we provide insight into the interplay between employees’ physical demands during work and off-job physical activity. We suggest that off-job physical activity will be particularly effective in preventing burnout for employees who face low (vs. high) physical job demands (Hobfoll et al. 2018; Meijman and Mulder 1998). We argue that whereas those with low physical job demands need physical activity to compensate for their physical *in*activity during the work day, those with high physical job demands cannot use the same functional system for work and recovery (Hobfoll et al. 2018; Meijman and Mulder 1998).

Third, we explore the relations between these various physical activities and burnout using a new conceptualization of burnout that supposedly aligns better with individuals’ daily experience of burnout. Specifically, we use a recently developed definition and associated assessment tool—the Burnout Assessment Tool (BAT; Schaufeli et al. 2020) and contribute to the burnout literature by expanding the BAT’s nomological network.

## Theoretical background

### Burnout

Traditionally, burnout is defined as a psychological syndrome unfolding as a prolonged response to chronic stressors on the job, consisting of exhaustion, a cynical attitude toward work, and a sense of professional inefficacy (Maslach and Leiter 2016). Over the past decades, this definition has received some criticism. For instance, Schaufeli and Taris (2005) showed that unwillingness (manifested as ‘cynicism’; Taris et al. 2005), and inability (manifested as ‘exhaustion’) are the two core burnout symptoms (see also, Demerouti et al. 2010). These authors proposed that professional inefficacy develops largely independently of exhaustion and cynicism and is most likely a consequence of these core symptoms. Further, Schaufeli and his colleagues (2020) have argued that the original burnout definition is incomplete. They reasoned that an inability to exert effort also involves emotional and cognitive impairments (inability to control emotions, inability to concentrate and focus), and such impairments are not considered in the most common definitions of burnout. Interestingly, the authors also consider secondary symptoms that are often reported by individuals who experience burnout, including depressed mood, psychological distress, and psychosomatic complaints. Since the Maslach Burnout Inventory (Maslach et al. 1997) cannot be used to identify burnout as an overall syndrome in clinical practice (Eurofound 2018), Schaufeli et al. (2020) proposed the burnout assessment tool for diagnosing burnout. They defined burnout as employees’ work-related state characterized by extreme tiredness, reduced ability to regulate cognitive and emotional processes, and mental distancing. Additionally, they included secondary burnout symptoms in this new definition (i.e., depressed mood and nonspecific psychological and psychosomatic distress symptoms) that often co-occur with the core burnout symptoms.

### Physical demands in relation to burnout

It is well known that high job demands pose an important risk of developing burnout (Alarcon 2011; Aronsson et al. 2017; Guthier et al. 2020). Job demands refer to those physical, psychological, social, or organizational aspects of the job that require sustained physical and/or psychological (cognitive and emotional) effort or skills (Demerouti et al. 2001). Therefore, job demands are associated with the activation of employees’ psychophysiological systems and result in physiological and psychological costs (Demerouti and Bakker 2011). When employees are highly and constantly exposed to job demands, their psychophysiological systems remain activated, physiological and psychological costs accumulate, and adverse effects, such as burnout, may arise (Bayes et al. 2021; Oerlemans and Bakker 2014).

In the present study, we suggest that *physical* job demands predict burnout. Physical job demands refer specifically to physical aspects of occupational tasks, such as heavy lifting, static and constrained working postures, and other (monotonous) physical activities required to perform work tasks. There is accumulating evidence that physical job demands have adverse physical and mental health effects (e.g., Coenen et al. 2020; Holtermann et al. 2018; Li et al. 2013). One crucial problem is that consistently high physical job demands may breach employees’ psychophysiological boundaries, which—over time—may lead to physical and mental health impairments. That is, physical job demands often involve occupational physical activity that is of low intensity and long duration combined with limited autonomy over how much and when to rest. Further, employees have little to say about the fit between their physical capacity and their physical demands (Holtermann et al. 2018). These demands are, by definition, hard to deal with and failure to meet the demands is likely (Schaufeli and Taris 2014). As a result, negative emotions, such as frustration, annoyance, and tension, may arise. These negative emotions need to be regulated (Gyurak et al. 2011) and may evoke feelings of emotional exhaustion and a tendency to mentally and physically withdraw from work (Boksem and Tops 2008). Moreover, it is conceivable that physical exhaustion has a reinforcing impact on mental exhaustion, as both experiences are hard to separate phenomenologically (Hockey 2013).

Little is known about the effects of physical job demands on mental health outcomes such as burnout (Cillekens et al. 2020). A possible reason is that it has been widely assumed that exposure to *cognitive* or *emotional* demands leads to burnout (Maslach and Leiter 2016). However, we argue that physical demands may also pose a risk. Research has shown that employees facing high physical job demands (i.e., blue-collar workers) can experience burnout symptoms too (Toppinen-Tannen et al. 2002). Furthermore, the few available studies show a positive relation between physical job demands and burnout (De Jonge et al. 2000; Schaufeli and Bakker 2004; Xanthopoulou et al. 2007). Given these previous empirical findings and based on the rationale that physical job demands overtax employees’ energy reservoir, we propose:

#### *Hypothesis 1: *

physical job demands are positively related to burnout.

### Physical activity as a resource-building activity

Whereas frequent exposure to high physical job demands may be taxing, we contend that off-job physical activity (i.e., during transportation, household chores and gardening, and recreation) may generate energetic, physical, and cognitive resources that help to prevent burnout. Compared to physical job demands, off-job physical activity is often of higher intensity and shorter duration, and individuals may match their physical activity to their physical capability (Holtermann et al. 2018). This opportunity to regulate makes it more likely that the activity builds resources instead of draining them. Off-job physical activity may help to replenish resources that have been used during the workday, ensuring that employees feel re-energized when facing a new workday (i.e., ‘recovery’; Sonnentag, 2003) and preventing the accumulation of physiological and psychological costs (Bayes et al. 2021). Further, off-job physical activity may help gain new additional personal resources that enable coping with future job demands (Demerouti et al. 2009; Steed et al. 2019; Ten Brummelhuis and Bakker 2012).

Off-job physical activity may build resources in various ways. First of all, off-job physical activity may improve employees’ physical resources (Caspersen et al. 1985; Chatterjee et al. 2019; Schmied et al. 2020), which is important to experience job demands as not overly fatiguing or stressful. Research shows that off-job physical activity elicits the neurophysiological stress system in a way that is similar to what psychosocial stressors do. When combined with sufficient bodily recovery, physical activity may cause physiological stress adaptation, resulting in faster physiological recovery after being exposed to stress (Klaperski et al. 2014; Landers and Arent 2007; Sothmann 2006). Furthermore, off-job physical activity may help to build cognitive resources, such as executive functions and memory (Fernandes et al. 2017; Zhu et al. 2017). This may help employees to restore cognitive resources that have been lost during the workday or be more resistant to unfavorable effects of cognitive job demands (Kulikowski 2020). Third, physical activity may also facilitate psychological detachment (not thinking about work outside work hours; Demerouti et al. 2009) because attention is drawn to bodily processes (Van Hooff et al. 2019). In this way, physical activity helps employees to take a cognitive respite from work stress (Radstaak et al. 2011) and aids recovery (Demerouti et al. 2009). Fourth, off-job physical activity may increase momentary and lasting positive affective states, such as happiness and relaxation (e.g., Basso and Suzuki 2017; Wiese et al. 2017), and decrease momentary and lasting negative affective states, such as stress and depression (e.g., Basso and Suzuki 2017; Dishman et al. 2021). Fifth, off-job physical activity may help to build social resources. Engaging in physical activity with others may provide social support and help build a stronger social network. Social support, in turn, may facilitate adaptive coping with stressful (work) situations and hence prevent burnout (Halbesleben 2006; Rueger et al. 2016). Finally, off-job physical activity is associated with mastery experiences and personal control over the environment, resulting in increased self-efficacy (Kandola et al. 2019). Self-efficacious employees are generally better able to cope with work tasks and job stressors.

Some off-job physical activities may be more strongly related to recovery and reduced burnout than other activities. All off-job physical activity types may help to distract from work-related thoughts and increase physical and cognitive resources. However, some off-job physical activity types have a more compulsory character (household physical activities, transportation) than others (recreational activities). Furthermore, some activities are chosen for enjoyment or perceived benefits (e.g., recreational activities and gardening). Research has shown that leisure activities especially aid recovery if the activity is voluntary, desirable, and enjoyable (Isoard-Gautheur et al. 2019; Oerlemans and Bakker 2014). Recreational physical activities may be best for recovery, as these often comprise challenging activities carried out together with significant others and provide excellent opportunities for mastery experiences and social support (Deci and Ryan 2000; Van den Broeck et al. 2008). Previous research has indeed shown that off-job physical activity is negatively related to burnout (Gerber et al. 2020; Naczenski et al. 2017). However, this research did not differentiate between different types of physical activity during non-work time. We explore such differential effects in the present study and propose that all types of physical activity during non-work time can alleviate job stress and burnout.

#### *Hypothesis 2:*

off-job physical activity (during transportation, household chores and gardening, and recreation) is negatively related to burnout.

### The combination of physical activities on and off the job

Although physical activity is hypothesized to have a favorable impact on burnout, we expect that this effect will not be the same for employees in all possible working conditions. Specifically, we argue that physical activity during non-work hours will be more helpful to reduce or prevent burnout complaints for employees with low versus high physical job demands. Our proposition is in line with the theoretical notion that recovery from job demands particularly occurs when the resources that are needed during work are no longer called upon during leisure time and that resources are notably regained when drawing on other resources than during work (Hobfoll et al. 2018; Meijman and Mulder 1998). Although off-job physical activity may build emotional, physical, and cognitive resources, it also causes short-term depletion of physical resources (e.g., muscle tissue damage, hormonal disturbances) (Graaf-Roelfsema et al. 2007). This means that when employees are physically active during work and leisure time on a daily basis, they constantly appeal to their physical resources, and daily recovery may be incomplete. After a day with high physical job demands and off-job physical activity, employees start the next day in a suboptimal condition. Therefore, they need to invest compensatory effort to cope with their physical job demands, resulting in an accumulation of physiological and psychological costs. In contrast, employees who are exposed to low physical job demands only use their physical resources to a limited extent during work time. These employees will benefit more from off-job physical activity because they can build new psychological and physical resources by drawing on other resources during non-work time than during work (cf. Hobfoll et al. 2018; Meijman and Mulder 1998).

Results of empirical studies investigating the impact of the combination of physical activity on and off the job on employee health are mixed. Some studies have shown that the presence of physical activities on and off the job increases the risk of impaired mental (Asztalos et al. 2009; Cerin et al. 2009) or physical health (Clays et al. 2013; Ferrario et al. 2018). Some studies show that both physical activities do not interact and work independently (Holtermann et al. 2021; Krause et al. 2017). Other studies have shown that off-job physical activity protects blue-collar workers’ physical health (Leino-Arjas et al. 2004; Quinn et al. 2021) or particularly white-collars’ level of burnout (Bernaards et al. 2006; Prince et al. 2021). To the best of our knowledge, no previous study has investigated the impact of the combination of physical activity on and off the job on burnout. However, studies in the sports domain show that endurance training combined with insufficient (bodily) rest periods—a situation that resembles high physical job demands combined with high off-job physical activity—is positively related to burnout (Gustafsson 2007). Since employees with high physical job demands will not optimally use off-job physical activity to restore and build resources, whereas employees with low physical job demands can restore and build resources during non-work time through physical activity, we predict:

#### *Hypothesis 3:*

the negative relationship between off-job physical activity and burnout is moderated by physical job demands. Specifically, this relationship is stronger for employees with low (vs. high) physical job demands.

## Method

### Procedure and participants

This study used a two-wave full panel design with a half-year time lag. The participants were recruited via the Website Amazon’s Mechanical Turk (MTurk; www.MTurk.com). Participants filled out two surveys (T1 & T2) in exchange for a monetary reward of $1 each. They were eligible to participate if they were currently employed and worked full-time (≥ 36 h a week). Only participants who filled out both surveys were included in the current study. A total of *n* = 848 only filled out the survey at T1, and *n* = 355 filled out the questionnaire at both T1 and T2. The final sample consisted of *N* = 355 participants. Of this final sample, most participants were male (*n* = 206, 58%) and relatively young (40.0% between 25 and 34 years old; 29.3% between 35 and 44 years old; 15.5% between 45 and 54 years old; < 7% in other categories). All participants were U.S. residents and were employed at the time of our measurements. Furthermore, most participants were highly educated (73% obtained at least a Bachelor’s degree). Participants had relatively high levels of autonomy (*M* = 3.23 [*SD* = 0.63] on a 4-point scale) and task demands (*M* = 2.93 [*SD* = 0.48] on a 4-point scale) at work. Lastly, participants evaluated their physical fitness as above average (*M* = 3.29 [*SD* = 0.77] on a 5-point scale).

### Dropout analysis

Participants who filled out both measurements reported less secondary burnout symptoms (psychosomatic and psychological complaints; *M* = 2.35, *SD* = 0.05, *F*(1, 831) = 5.80, *p* = 0.02, η^2^ = 0.007) at baseline than participants who only filled out the first measurement (*M* = 2.50, *SD* = 0.04), but did not differ on other baseline outcomes.

### Materials

**Off-job physical activity.** Three types of off-job physical activity were measured: (a) transportation (traveling by public transport, bicycling, and walking from place to place); (b) household (vigorous and moderate housework, gardening, yard work, general maintenance work, and caring for family); and (c) recreational (vigorous and moderate physical activity for recreation, sport, exercise or leisure, and walking) using 18 items of the long version of the ‘International Physical Activity Questionnaire’ (IPAQ) by Hagströmer and colleagues (2006). Participants were asked about their engagement in physical activity during the last 7 days by indicating how many days and the average duration per day they engaged in a specific type of physical activity. An example item is “Think about only those physical activities that you did for at least 10 min at a time. During the last seven days, on how many days did you do vigorous physical activities like heavy lifting, chopping wood, shoveling snow, or digging in the garden or yard?” Participants could answer by indicating the number of days a week. After this item, the following question was asked: “How much time did you usually spend on one of those days doing vigorous physical activities in the garden or yard?” Participants could indicate the average hours/minutes per day they engage in this activity. In the current investigation, the weekly MET minutes of physical activity was used as an outcome variable. One MET is a multiple of the estimated resting energy expenditure (Forde 2018). MET minutes were calculated by multiplying the MET value given for an activity (walking = 3.3 METs, moderate activity = 4.0 METs, and vigorous activity = 8.0 METs) by the minutes the activity was carried out and the number of days that the activity was undertaken. We followed the IPAQ scoring protocol recommendations for data cleaning and processing (Patterson 2005). Previous research indicates that the IPAQ has acceptable measurement properties with a reliability of 0.80 (Hallal and Victora 2004) and a re-test reliability of 0.70 (Craig et al. 2003). As the IPAQ scores had skewed distributions, we log-transformed these scores before statistical analysis.

**Burnout.** The general version of the Burnout Assessment Tool (BAT) by Schaufeli et al. (2020) was utilized to assess the presence of core (i.e., exhaustion, mental distance, cognitive impairment, and emotional impairment) and secondary (i.e., psychological and psychosomatic complaints) burnout symptoms with 32 items that could be scored on a five-point Likert scale ranging from *never* (1) to *always* (5). Example items are ‘I feel mentally exhausted’ and ‘I feel unable to control my emotions’. Two average scores (for core and secondary burnout symptoms) were used as outcome variables. Previous research shows that the General version of the BAT has a test–retest reliability of 0.74 (Schaufeli et al. 2020). Internal reliability of core burnout symptoms (T1: α = 0.87; T2: α = 0.87) and secondary burnout symptoms (T1: α = 0.92; T2: α = 0.92) was high in the present study.

**Physical demands.** Physical demands were assessed with five items of the Job Content Questionnaire (Karasek et al. 1998). Items could be answered on a 4-point Likert scale, ranging from 1 (*almost never*) to 4 (*almost always*). An example item is: ‘Does your work require rapid continuous physical activity?’ and ‘Are you required to move or lift very heavy loads in your job?’ Internal reliability was good (T1: α = 0.89; T2: α = 0.89).

**Control variables.** Age, gender and educational level were included as control variables, as these variables has been shown to be related to burnout (Brewer and Shapard 2004; Hakanen et al. 2011; Purvanonva and Muros 2010).

### Statistical approach

Hierarchical regression analyses were carried out to test our hypotheses. For Hypotheses 1 and 2, two models were computed, separately for primary and secondary burnout symptoms. The first model included the control variables (age, gender, educational level). The second model included the control variables and predictor variables of interest (respectively, physical job demands [H1] and the three types of off-job physical activity [H2]). Further, three models were computed to test Hypothesis 3. The first model contained the control variables, the second model the control and predictor variables (physical job demands and the three types of off-job physical activity), and in the third model the three interaction terms were added (between physical job demands and each off-job physical activity type). When a significant interaction term was found, the Johnson–Neyman technique was used to interpret the interaction (Carden et al. 2017). The Johnson–Neyman technique allows for identifying the regions in the range of the moderator variable where the effect of the predictor on the outcome is statistically significant.

## Results

### Descriptive statistics

Table 1 displays the means (*M*), standard deviations (*SD*), and correlations (*r)* of all outcome variables. As can be seen in Table 1, burnout remained very stable between T1 and T2, as indicated by a correlation of 0.81 for core and 0.74 for secondary symptoms. As these large auto-correlations do not leave much variance to be explained, we report prospective results (X at T1 Y at T2) in this results section. The results of cross-sectional analyses (X–Y relations tested at one time point, T1 or T2) and longitudinal analyses (X at T1 Y at T2, controlled for Y at T1) can be found in the Supplementary Material.

**Table 1:** Correlation table

	*M* (*SD*)	1	2	3	4	5	6	7	8	9	10	11	12	13	14
1.Gender	n/a														
2. Age	n/a	0.16**													
3.Educational level	n/a	− 0.10	− 0.30*												
4.Physical job demands T1	2.01 (0.90)	− 0.03	− 0.18*	< 0.01											
5. Physical job demands T2	2.04 (0.88)	− 0.10	− 0.25*	0.04	0.72**										
6. Burnout core symptoms T1	2.48 (0.86)	− 0.09	− 0.40**	0.09	0.39**	0.30**									
7. Burnout core symptoms T2	2.52 (0.87)	− 0.13*	− 0.39**	0.09	0.32**	0.37**	0.81**								
8. Burnout secondary symptoms T1	2.35 (0.87)	< 0.01	− 0.34**	0.04	0.43**	0.35**	0.83**	0.68**							
9. Burnout secondary symptoms T2	2.40 (0.88)	− 0.02	− 0.30**	0.02	0.35**	0.41**	0.70**	0.81**	0.74**						
10. Transportation off-job PA T1	484.68 (610.27)	− 0.13*	− 0.15**	0.22**	0.15**	0.18**	0.16**	0.16**	0.10	0.14**					
11. Transportation off-job PA T2	502.01 (835.22)	− 0.16**	− 0.12**	0.16**	0.06	0.13**	0.08	0.04	0.07	0.07	0.42**				
12. Household off-job PA T1	1040.16 (1320.51)	0.06	0.13*	0.07	0.07	0.07	− 0.04	− 0.06	− 0.04	< 0.01	0.21**	0.16**			
13. Household off-job PA T1	986.20 (1156.95)	0.08	0.11*	− 0.03	0.08	0.08	− 0.01	− 0.03	0.03	0.02	0.06	0.30**	0.33**		
14. Recreational off-job PA T1	1036.92 (1368.81)	− 0.08	− 0.10	0.19**	0.10	0.09	0.01	0.03	− 0.02	0.03	0.25**	0.25**	0.31**	0.09	
15. Recreational off-job PA T2	947.03 (1221.30)	− 0.07	− 0.05	0.16**	0.08	0.08	0.06	0.02	0.03	0.01	0.21**	0.44**	0.22**	0.44**	0.43**

*p < 0.01, **p < 0.001

### Physical job demands predicting burnout

See Table 2 for the results of testing Hypothesis 1 (physical job demands are positively related to burnout). The first model shows that the control variables accounted for a significant amount of variance in core and secondary burnout symptoms. Age turned out to be a significant predictor of core (β = − 0.38) and secondary (β = − 0.33) burnout symptoms—older employees were less likely to experience these symptoms. Gender and educational level were not related to core and secondary burnout symptoms. In the second model, physical job demands were entered into the regression models, which showed a significant improvement of the models for both core and secondary symptoms. Results revealed that physical job demands were positively related to core (β = 0.26) and secondary (β = 0.31) burnout symptoms. This means that Hypothesis 1 is supported prospectively. The positive associations were also found cross-sectionally (at T1 or at T2). However, the positive associations disappeared when controlling for previous levels of the outcome variables (see Supplementary Material).

**Table 2:** Prospective regression models predicting core and secondary burnout symptoms at T2 from physical job demands at T1

	Core burnout symptoms (T2)						Secondary burnout symptoms (T2)					
	Model 1			Model 2			Model 1			Model 2		
	*b*	*SE*	*p*	*b*	*SE*	*P*	*b*	SE	*p*	*b*	SE	*p*
Constant	3.71	0.30	< 0.001	3.04	0.31	< 0.001	3.37	0.31	< 0.001	2.57	0.32	< 0.001
Gender	− 0.13	0.09	0.13	− 0.13	0.08	0.13	0.05	0.09	0.58	0.06	0.09	0.52
Age	− 0.30	0.04	< 0.001	− 0.26	0.04	< 0.001	− 0.26	0.04	< 0.001	− 0.22	0.04	< 0.001
Educational level	− 0.05	0.08	0.51	− 0.03	0.08	0.68	− 0.11	0.08	0.18	− 0.09	0.08	0.28
Physical job demands T1					0.25	< 0.001				0.30	0.05	< 0.001
*F*	21.38			24.44			12.63			20.24		
*R*^*2*^	0.15			0.22			0.10			0.19		
Adjusted *R*^*2*^	0.15			0.21			0.09			0.18		
Δ*R*^*2*^				0.06			0.10			0.09		

### Off-job physical activities predicting burnout

See Table 3 for the results to test Hypothesis 2 (the three types of off-job physical activity [transportation, household, and recreation physical activity] are negatively related to burnout). In the first step, control variables were included. The second model, including the addition of the three types of off-job physical activity, showed no significant improvement of the first model for both core and secondary burnout symptoms (see ΔR^2^ in Table 3). Contrary to expectations, in the second model, transportation physical activity was positively related to core (β = 0.11*)* and secondary (β = 0.12) burnout symptoms. Household and recreation physical activity were not related to core (respectively β = − 0.03 and β = − 0.02) and secondary (respectively β = 0.03 and β = − 0.02) burnout symptoms. This means that Hypothesis 2 is not supported. The positive association between transportation physical activity and core burnout symptoms was also found at T1 (cross-sectionally), but not at T2 (cross-sectionally) or longitudinally, and not for secondary burnout symptoms (see Supplementary Material).

**Table 3:** Prospective regression models predicting core and secondary burnout symptoms at T2 from the different types of off-job physical activity at T1

	Core burnout symptoms (T2)						Secondary burnout symptoms (T2)					
	Model 1			Model 2			Model 1			Model 2		
	*b*	*SE*	*p*	*b*	*SE*	*P*	*b*	SE	*p*	*b*	SE	*p*
Constant	3.71	0.30	<0 .001	3.63	0.31	< 0.001	3.37	0.31	< 0.001	3.22	0.32	< 0.001
Gender	− 0.13	0.09	0.13	− 0.11	0.09	0.13	0.05	0.09	0.58	0.07	0.09	0.45
Age	− 0.30	0.04	< 0.001	− 0.29	0.04	< 0.001	− 0.26	0.04	< 0.001	− 0.26	0.04	< 0.001
Educational level	− 0.05	0.08	0.51	− 0.08	0.08	0.35	− 0.11	0.08	0.18	− 0.15	0.08	0.08
Transportation physical activity T1				0.04	0.02	0.03				0.04	0.02	0.03
Household physical activity T1				− 0.01	0.02	0.60				0.01	0.02	0.64
Recreation physical activity T1				− 0.01	0.02	0.74				− 0.01	0.02	0.75
*F*	21.38			11.51			12.63			7.34		
*R*^*2*^	0.15			0.17			0.10			0.11		
Adjusted *R*^*2*^	0.15			0.15			0.09			0.10		
Δ*R*^*2*^				0.01						0.02		

### Interactions of off-job physical activities and physical job demands with burnout

**Fig. 1:**
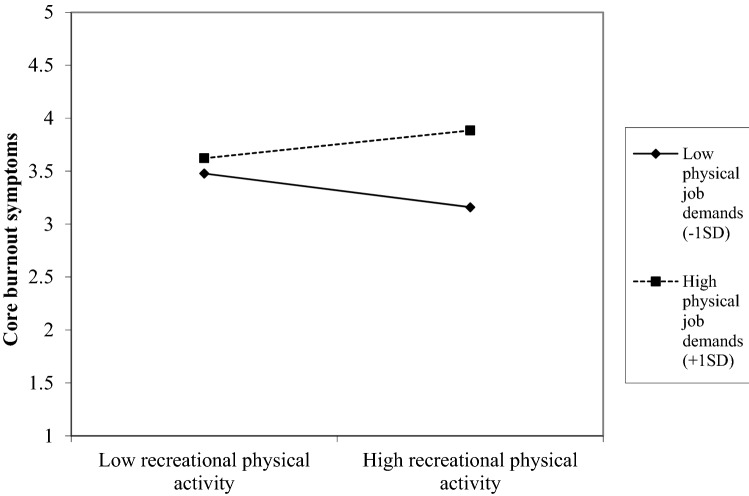
Physical job demands as a moderator in the relation between recreational physical activity and core burnout symptoms

**Fig. 2:**
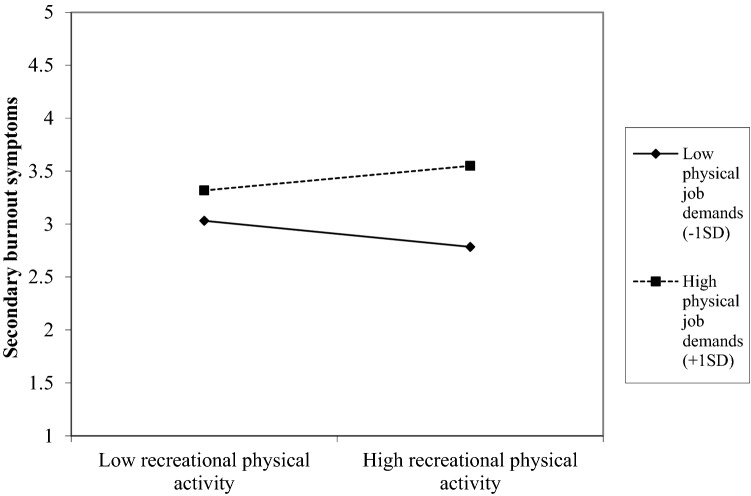
Physical demands as a moderator in the relation between recreational physical activity and secondary burnout symptoms

Results of testing Hypothesis 3 (the negative relation between off-job physical activity and burnout is stronger for employees with low vs. high physical job demands) can be found in Table 4. The second model, including the predictor variables (physical job demands and the three types of off-job physical activity), shows a significant improvement of the first model that only included control variables for both core and secondary burnout symptoms. In these second models, physical job demands significantly predicted burnout, and transportation, household, and recreational physical activity were unrelated to burnout. The third model, including the interaction terms between physical job demands and each of the three types of off-job physical activity, was significantly better than the second model for core burnout but not for secondary burnout symptoms (see ΔR^2^ in Table 4). Results revealed no interaction between physical job demands and transportation and household physical activity on core and secondary burnout symptoms. However, the interactions between physical job demands and recreational physical activity significantly predicted core and secondary burnout symptoms. These significant interactions for core burnout symptoms are illustrated in Fig. 1 and for secondary burnout symptoms in Fig. 2. The Johnson–Neyman technique (Carden et al. 2017) showed that the relationship between recreational physical activity and core burnout symptoms was significant and negative when physical job demands were low (lower than *M* = 1.36 on a 4-point scale), and significant and positive when physical job demands were high (higher than *M* = 3.15 on a 4-point scale). Similarly, the Johnson–Neyman technique revealed that the relationship between recreational physical activity and secondary burnout symptoms was significant and negative when physical job demands were low (lower than *M* = 1.35 on a 4-point scale), and significant and positive when physical job demands were high (higher than *M* = 2.73 on a 4-point scale). Given that the interplay between recreational physical activity and physical job demands was significant in predicting burnout, but physical job demands did not interact with transportation and household physical activity in predicting burnout, we conclude that Hypothesis 3 is partly supported. The interaction between physical job demands and recreational physical activity was also found cross-sectionally (at T1 or at T2). However, the interaction disappeared when controlling for previous levels of the outcome variables. Furthermore, a significant interaction between household physical activity and physical job demands was found at T2, suggesting a buffering effect of household physical activity on the positive relation between physical job demands and burnout (see Supplementary Material).

**Table 4:** Prospective regression models predicting core and secondary burnout symptoms at T2 from the interaction between physical job demands and different types of off-job physical activity at T1

	Core burnout symptoms (T2)									Secondary burnout symptoms (T2)								
	Model 1			Model 2			Model 3			Model 1			Model 2			Model 3		
	*b*	*SE*	*p*	*b*	*SE*	*p*	*b*	*SE*	*p*	*b*	SE	*p*	*b*	SE	*P*	*b*	SE	*p*
Constant	3.71	0.30	< 0.001	3.04	0.32	< 0.001	3.78	0.44	< 0.001	3.37	0.31	< 0.001	2.52	0.33	< 0.001	2.98	0.45	< 0.001
Gender	− 0.13	0.09	0.13	− 0.11	0.09	0.18	− 0.12	0.08	0.16	0.05	0.09	0.58	0.07	0.09	0.45	0.07	0.09	0.44
Age	− 0.30	0.04	< .001	− 0.26	0.04	< 0.001	− 0.25	0.04	< 0.001	− 0.26	0.04	< 0.001	− 0.21	0.04	< 0.001	− 0.21	0.02	< 0.001
Educational level	− 0.05	0.08	0.51	− 0.04	0.08	0.62	− 0.04	0.08	0.58	− 0.11	0.08	0.18	− 0.10	0.08	0.20	− 0.11	0.08	0.16
Transportation physical activity T1				0.03	0.02	0.10	0.01	0.04	0.88				0.03	0.02	0.09	0.00	0.04	0.94
Household physical activity T1				− 0.02	0.02	0.40	− 0.04	0.05	0.41				0.01	0.02	0.89	0.01	0.05	0.82
Recreation physical activity T1				− 0.01	0.02	0.54	− 0.11	0.03	< 0.01				− 0.01	0.02	0.52	− 0.09	0.04	0.02
Physical job demands T1				0.24	0.05	< 0.001	− 0.10	0.15	0.51				0.29	0.05	< 0.001	0.08	0.16	0.63
Transportation physical activity T1 x Physical job demands T1							0.01	0.02	0.71							0.01	0.02	0.56
Household physical activity T1 x Physical job demands T1							0.01	0.02	0.79							− 0.01	0.02	0.69
Recreational physical activity T1 x Physical demands T1							0.05	0.02	< 0.01							0.04	0.02	0.02
*F*	21.38			14.47			11.69			12.63			12.02			9.23		
*R*^*2*^	0.15			0.23			0.25			0.10			0.20			0.21		
Adjusted *R*^*2*^	0.15			0.21			0.23			0.09			0.18			0.19		
Δ*R*^*2*^				0.07			0.03						0.10			0.02

## Discussion

This study aimed to investigate different domain-specific physical activities (work vs. leisure time) in relation to burnout. To better align with the experience of burnout in practice, we used a recent new conceptualization and assessment of burnout (Schaufeli et al. 2020). We found that physical job demands and transportation physical activity were positively related to burnout symptoms. Household physical activity was unrelated to burnout symptoms. Further, we found that recreational physical activity was only negatively related to burnout symptoms among employees with low physical job demands. In contrast, recreational physical activity was related to more burnout symptoms among employees with high physical job demands.

### Theoretical implications

The present study has several theoretical implications. First, our research contributes to the literature by showing that a distinction between different domain-specific physical activities is relevant for predicting burnout. In contrast to earlier notions that physical activity may prevent and reduce burnout (Gerber et al. 2020; Naczenski et al. 2017; Ochentel et al. 2018), we showed that specific physical activities, i.e., physical job demands and transportation physical activity, were *positively* related to burnout symptoms. The positive association between physical job demands and burnout is in line with the ‘physical activity health paradox’ stating that physical activity at work impairs health, whereas off-job physical activity promotes it (Coenen et al. 2020; Holtermann et al. 2018). It is important to note that previous research on this paradox mainly focused on the adverse effects of physical activity at work on *physical* health (Cillekens et al. 2020). We showed that these adverse effects may also apply to burnout. The finding that transportation physical activity was positively related, and household physical activity was unrelated to burnout were contrary to our expectations and the ‘physical activity health paradox’ (Coenen et al. 2020; Holtermann et al. 2018). It is conceivable that the beneficial effects of the different types of off-job physical activity are only visible under certain circumstances. For instance, transportation physical activity could be a source of stress when it is compulsory (Isoard-Gautheur et al. 2019) or is carried out in an environment with noise, pollution, or a poor infrastructure (Asztalos et al. 2009; Chatterjee et al. 2019). Regarding household physical activity, it is possible that these activities simultaneously facilitate recovery (e.g., through the distraction of work) and hamper relaxation and mastery (e.g., because of their obligatory and repetitive character), which may result in null findings.

A second contribution is that we showed that certain physical activities interact in predicting burnout. It was demonstrated that recreational physical activity was only related to fewer burnout symptoms among employees with low physical job demands. Moreover, recreational physical activity was related to more burnout symptoms among employees with high physical job demands. These findings suggest that the simultaneous enactment of different physical activities alters their potential unique effects (cf. Prince et al. 2021). Therefore, researchers should not restrict themselves to one domain-specific physical activity when studying possible positive effects following physical activity. The results are in line with theoretical notions that recovery from job demands mainly occurs when the resources that are needed during work are no longer called upon during leisure time and that resources are notably regained when drawing on other resources than during work (Hobfoll et al. 2018; Meijman and Mulder 1998). Furthermore, our findings are in line with the contention that physical activity causes short-term depletion of physical resources (e.g., muscle tissue damage, hormonal disturbances; Graaf-Roelfsema et al. 2007) and—when it is not combined with sufficient bodily recovery—may result in an accumulation of physiological and psychological costs, eventually resulting in adverse outcomes such as burnout (see Demerouti et al. 2009).

Third, by showing that different physical activities and their interplay with physical job demands contribute to burnout, we expand the nomological network of a new definition of burnout (Schaufeli et al. 2020). These findings confirm previous suggestions and findings that non-work factors could be antecedents of burnout (Bianchi et al. 2021). Furthermore, the finding that the relations between the different physical activities and burnout did not differ based on whether core or secondary symptoms were included in the analyses is consistent with the notion that secondary burnout symptoms such as depressive and psychosomatic complaints are important indicators of burnout (Schaufeli et al. 2020). Further, whereas previous studies mainly showed that prolonged exposure to *cognitive* or *emotional* job demands leads to burnout (Maslach and Leiter 2016), we showed that exposure to *physical* job demands might lead to burnout as well. Just like cognitive and emotional job demands, physical job demands presumably contribute to sustained activation of physiological stress systems (e.g., autonomic nervous system and adrenal medullary axis; see Bayes et al. 2021), which may result in burnout (Landers and Arent 2007; Sothmann 2006).

### Strengths, limitations, and suggestions for future research

This study has several strengths. We used a time-lagged design that provided opportunities for testing relations and temporal precedence (the ‘cause’ should occur before the ‘outcome’), which are both criteria of determining causality (Spector 2019). Additionally, this study is high in ecological validity, as we used a new conceptualization and assessment of burnout that closely matches the daily burnout experience (Schaufeli et al. 2020). Finally, we incorporated various domain-specific physical activity types, which gives a more fine-grained understanding of how physical activity and burnout are related.

Despite the study’s strengths, several limitations of this study need to be addressed. First, we cannot make causal claims. We found that core and secondary burnout symptoms were highly stable over a half year. This stability implies that it is statistically speaking rather challenging to detect across-time relations. Indeed, we found that physical activities and burnout were cross-sectionally and prospectively related, but these relations disappeared when investigated longitudinally (i.e., when we controlled for the outcome at baseline). Thus, we could not find evidence for the idea that physical activity is related to a *change* in burnout. Earlier studies revealed that cross-lagged effects on burnout occurred at longer time intervals (see e.g., De Vries et al. 2016; Naczenski et al. 2017; Guthier et al. 2020). Accordingly, we suggest that future research adopts a more extended time lag (≥ 1 year) to study physical activity and burnout over time. Furthermore, if possible, including more than two measurement occasions is desirable to maximize the chances of finding the appropriate time lag (Guthier et al. 2020; Spector 2019).

Second—related to the previous limitation—we were not able to control for all relevant third variables, which is essential to allow for more robust causal inferences (Spector and Brannick 2011). For instance, we selected full-time working employees, but did not measure participants’ actual work hours. Previous research has shown that excessive work hours and working overtime are negatively related to off-job physical activity (Kirk and Rhodes 2011) and positively to burnout (Rabenu and Aharoni-Goldenberg 2017). Work hours may thus have acted as a potential third variable. Accordingly, in future research, we suggest incorporating these variables as controls (see Spector and Brannick 2011 for suggestions on how to do this).

The third limitation concerns our physical activity measure. Physical activity was self-reported. Although this measure has been found to be reliable and valid (Craig et al. 2003; Hallal and Victora 2004), research has also shown that people sometimes find it difficult to adequately recall their actual physical activity level (Dowd et al. 2018). Furthermore, our used measure did not explicitly ask for strength training (i.e., it was asked how much time/how many days participants engaged in moderate and vigorous activities, and some examples of these activities were provided), while previous research indicates that this type of physical activity may have health benefits among employees with physically demanding work (Sundstrup et al. 2020). Preferably, future research should include device-based measures such as accelerometers or specifically ask for strength training to better assess physical activity (Skotte et al. 2014).

The fourth limitation relates to the generalizability of our findings. We studied full-time working employees (US residents) who were also enrolled in Amazon’s MTurk. Although previous research concludes that MTurk samples are a viable source for occupational health research (Michel et al. 2018; Keith et al. 2017), it has been shown that MTurk samples tend to be younger, more educated, have lower income, engage in less healthy behaviors, and have more mental health problems than nationally representative samples (Keith et al. 2017; Walters et al. 2018). Further, white-collar employees (especially employed in technological jobs) seem to be slightly overrepresented compared to blue-collar employees (Michel et al. 2018). A positive point is that MTurk samples seem to come from a more diverse set of industries than samples typically used in occupational health research (Michel et al. 2018; Keith et al. 2017). To increase generalizability, we suggest that future research replicate our findings using representative samples of the general population, preferably including more blue-collar workers.

Fifth, it is possible that attrition bias has played a role in this study, since participants who dropped out at the second measurement reported more secondary burnout symptoms at baseline (medium effect size). This systematic attrition might have resulted in a restriction of range in burnout and an underestimation of the found relations (Asendorpf et al. 2014). Unfortunately, attrition is very common in longitudinal studies. We suggest that future research tries to limit attrition or use multiple imputations when data are missing at random (see e.g., Asendorpf et al. 2014).

### Practical implications

Our research has several practical implications. We found that full-time workers exposed to physical job demands are at higher risk for burnout. This result and the previously found adverse health effects of physically demanding work (Coenen et al. 2020; Holtermann et al. 2018) highlight the importance of organizational preventive measures such as supervisor support (Clays et al. 2016), decision authority, skill discretion (Viotti et al. 2017), workplace strength training (Sundstrup et al. 2020), and exoskeletons (Shepertycky et al. 2021) to buffer the negative effects of high physical job demands. Further, to prevent incomplete recovery from work, it seems advisable that full-time working employees with physically demanding work do not overly engage in recreational physical activities during non-work time. Possibly, these employees could benefit from other recovery strategies that do not require sustained physical effort, such as social and relaxation activities (Sonnentag et al. 2017). In contrast, for full-time working employees with low physical demands (white-collar workers), it seems advisable to engage in recreational physical activity to enhance work recovery and prevent burnout.

## Conclusion

This study shows that physical activities on and off the job interact and do not play the same role in preventing burnout. Our findings point to a physical activity paradox in which physically demanding work is related to more burnout symptoms, and that recreational physical activity strengthens this relationship. In contrast, recreational physical activity is related to fewer burnout symptoms among employees who do not have physically demanding work. These findings suggest that employees’ off-job physical activity should be tailored based on employees’ level of physical activity at work to lower the risk of job burnout. To allow for firmer causal inferences regarding the relations between different physical activities and burnout, we suggest that future longitudinal investigations use longer time intervals between measurement points, control for relevant third variables, use device-based physical activity measures, and try to limit systematic attrition.

## Supplementary Information

Below is the link to the electronic supplementary material.

Supplementary file1 (DOCX 57 KB)

## Data Availability

Data and material can be requested from the corresponding author.
