# Supplementary material for: The physical activity paradox: a longitudinal study of the implications for burnout
Source: Int Arch Occup Environ Health. 2021 Oct 6;95(5):965–79. doi: 10.1007/s00420-021-01759-y (PMC9203402; doi:10.1007/s00420-021-01759-y)
Supplement: Supplementary file 1 — Supplementary file1 (DOCX 57 KB) [file 420_2021_1759_MOESM1_ESM.docx]

**Supplementary material**

**Testing Hypothesis 1 Cross-sectionally and longitudinally**

Table 1A. *Cross-sectional regression models predicting core and secondary burnout symptoms at T1 from physical job demands at T1.*

|  | Core burnout symptoms (T1) | | | | | | Secondary burnout symptoms (T1) | | | | | |
| --- | --- | --- | --- | --- | --- | --- | --- | --- | --- | --- | --- | --- |
|  | Model 1 | | | Model 2 | | | Model 1 | | | Model 2 | | |
|  | *b* | *SE* | *p* | *b* | *SE* | *p* | *b* | SE | *p* | *b* | SE | *p* |
| Constant | 3.56 | .30 | <.001 | 2.70 | .30 | <.001 | 3.34 | .31 | <.001 | 2.36 | .31 | <.001 |
| Gender | -.06 | .09 | .50 | -.05 | .08 | .51 | .09 | .09 | .32 | .10 | .08 | .25 |
| Age | -.31 | .04 | <.001 | -.26 | .04 | <.001 | -.29 | .04 | <.001 | -.24 | .04 | <.001 |
| Educational level | -.04 | .08 | .58 | -.02 | .04 | .84 | -.11 | .08 | .19 | -.08 | .07 | .31 |
| Physical job demands T1 |  |  |  |  | .32 | <.001 |  |  |  | .37 | .05 | <.001 |
| *F* | 22.03 |  |  | 31.73 |  |  | 16.56 |  |  | 30.98 |  |  |
| *R^2^* | .16 |  |  | .27 |  |  | .12 |  |  | .26 |  |  |
| Adjusted *R^2^* | .15 |  |  | .26 |  |  | .12 |  |  | .25 |  |  |
| Δ*R^2^* |  |  |  | .11 |  |  |  |  |  | .14 |  |  |

Table 1B. *Cross-sectional regression models predicting core and secondary burnout symptoms at T2 from physical job demands at T2.*

|  | Core burnout symptoms (T2) | | | | | | Secondary burnout symptoms (T2) | | | | | |
| --- | --- | --- | --- | --- | --- | --- | --- | --- | --- | --- | --- | --- |
|  | Model 1 | | | Model 2 | | | Model 1 | | | Model 2 | | |
|  | *b* | *SE* | *p* | *b* | *SE* | *p* | *b* | SE | *p* | *b* | SE | *p* |
| Constant | 3.71 | .30 | <.001 | 2.86 | .32 | <.001 | 3.37 | .31 | <.001 | 2.31 | .32 | <.001 |
| Gender | -.13 | .09 | .13 | -.10 | .08 | .22 | .05 | .09 | .58 | .09 | .09 | .29 |
| Age | -.30 | .04 | <.001 | -.24 | .04 | <.001 | -.26 | .04 | <.001 | -.19 | .04 | <.001 |
| Educational level | -.05 | .08 | .51 | -.03 | .08 | .67 | -.11 | .08 | .18 | -.09 | .08 | .27 |
| Physical job demands T2 |  |  |  | .28 | .05 | <.001 |  |  |  | .36 | .05 | <.001 |
| *F* | 21.38 |  |  | 26.55 |  |  | 12.63 |  |  | 24.34 |  |  |
| *R^2^* | .15 |  |  | .23 |  |  | .10 |  |  | .22 |  |  |
| Adjusted *R^2^* | .15 |  |  | .22 |  |  | .09 |  |  | .21 |  |  |
| Δ*R^2^* |  |  |  | .08 |  |  |  |  |  | .12 |  |  |

Table 1C. *Longitudinal regression models predicting core and secondary burnout symptoms at T2 from physical job demands at T1, controlled for burnout symptoms at T1.*

|  | Core burnout symptoms (T2) | | | | | | Secondary burnout symptoms (T2) | | | | | |
| --- | --- | --- | --- | --- | --- | --- | --- | --- | --- | --- | --- | --- |
|  | Model 1 | | | Model 2 | | | Model 1 | | | Model 2 | | |
|  | *b* | *SE* | *p* | *b* | *SE* | *p* | *b* | SE | *p* | *b* | SE | *p* |
| Constant | 3.71 | .30 | <.001 | .93 | .23 | <.001 | 3.37 | .31 | <.001 | *.*90 | .26 | <.001 |
| Gender | -.13 | .09 | .13 | -.09 | .06 | .12 | .05 | .09 | .58 | -.01 | .06 | .86 |
| Age | -.30 | .04 | <.001 | -.06 | .03 | .04 | -.26 | .04 | <.001 | -.05 | .03 | .14 |
| Educational level | -.05 | .08 | .51 | -.02 | .05 | .71 | -.11 | .08 | .18 | -.03 | .06 | .58 |
| Burnout symptoms at T1 |  |  |  | .78 | .04 | <.001 |  |  |  | .71 | .04 | <.001 |
| Physical job demands T1 |  |  |  | <.01 | .03 | .98 |  |  |  | .04 | .04 | .31 |
| *F* | 21.38 |  |  | 133.83 |  |  | 12.63 |  |  | 87.19 |  |  |
| *R^2^* | .15 |  |  | .66 |  |  | .10 |  |  | .56 |  |  |
| Adjusted *R^2^* | .15 |  |  | .65 |  |  | .09 |  |  | .55 |  |  |
| Δ*R^2^* |  |  |  | .50 |  |  |  |  |  | .46 |  |  |

**Testing Hypothesis 2 Cross-sectionally and longitudinally**

Table 2A. *Cross-sectional regression models predicting core and secondary burnout symptoms at T1 from the different types of off-job physical activity at T1.*

|  | Core burnout symptoms (T1) | | | | | | Secondary burnout symptoms (T1) | | | | | |
| --- | --- | --- | --- | --- | --- | --- | --- | --- | --- | --- | --- | --- |
|  | Model 1 | | | Model 2 | | | Model 1 | | | Model 2 | | |
|  | *b* | *SE* | *p* | *b* | *SE* | *p* | *b* | SE | *p* | *b* | SE | *p* |
| Constant | 3.56 | .30 | <.001 | 3.47 | .31 | <.001 | 3.34 | .31 | <.001 | 3.30 | .32 | <.001 |
| Gender | -.06 | .09 | .50 | -.04 | .09 | .62 | .09 | .09 | .32 | .10 | .09 | .28 |
| Age | -.31 | .04 | <.001 | -.31 | .04 | <.001 | -.29 | .04 | <.001 | -.29 | .04 | <.001 |
| Educational level | -.04 | .08 | .58 | -.06 | .08 | .42 | -.11 | .08 | .19 | -.12 | .08 | .16 |
| Transportation physical activity T1 |  |  |  | .04 | .02 | .02 |  |  |  | .03 | .02 | .12 |
| Household physical activity T1 |  |  |  | <.01 | .02 | .82 |  |  |  | <.01 | .02 | .84 |
| Recreation physical activity T1 |  |  |  | -.02 | .02 | .26 |  |  |  | -.02 | .02 | .23 |
| *F* | 22.03 |  |  | 12.14 |  |  | 16.56 |  |  | 8.84 |  |  |
| *R^2^* | .16 |  |  | .17 |  |  | .12 |  |  | .13 |  |  |
| Adjusted *R^2^* | .15 |  |  | .16 |  |  | .12 |  |  | .12 |  |  |
| Δ*R^2^* |  |  |  | .02 |  |  |  |  |  | .01 |  |  |

Table 2B. *Cross-sectional* *regression models predicting core and secondary burnout symptoms at T2 from the different types of off-job physical activity at T2.*

|  | Core burnout symptoms (T2) | | | | | | Secondary burnout symptoms (T2) | | | | | |
| --- | --- | --- | --- | --- | --- | --- | --- | --- | --- | --- | --- | --- |
|  | Model 1 | | | Model 2 | | | Model 1 | | | Model 2 | | |
|  | *b* | *SE* | *p* | *b* | *SE* | *p* | *b* | SE | *p* | *b* | SE | *p* |
| Constant | 3.71 | .30 | <.001 | 3.70 | .31 | <.001 | 3.37 | .31 | <.001 | 3.26 | .33 | <.001 |
| Gender | -.13 | .09 | .13 | -.14 | .09 | .11 | .05 | .09 | .58 | .05 | .09 | .58 |
| Age | -.30 | .04 | <.001 | -.31 | .04 | <.001 | -.26 | .04 | <.001 | -.26 | .04 | <.001 |
| Educational level | -.05 | .08 | .51 | -.05 | .08 | .55 | -.11 | .08 | .18 | -.11 | .08 | .18 |
| Transportation physical activity T2 |  |  |  | -.01 | .02 | .63 |  |  |  | .01 | .02 | .49 |
| Household physical activity T2 |  |  |  | .01 | .02 | .69 |  |  |  | .02 | .02 | .34 |
| Recreation physical activity T2 |  |  |  | <.01 | .02 | .95 |  |  |  | -.01 | .02 | .59 |
| *F* | 21.38 |  |  | 10.67 |  |  | 12.63 |  |  | 6.57 |  |  |
| *R^2^* | .15 |  |  | .16 |  |  | .10 |  |  | .10 |  |  |
| Adjusted *R^2^* | .15 |  |  | .14 |  |  | .09 |  |  | .09 |  |  |
| Δ*R^2^* |  |  |  | <.01 |  |  |  |  |  | <.01 |  |  |

Table 2C. *Longitudinal regression models predicting core and secondary burnout symptoms at T2 from the different types of off-job physical activity at T1, controlled for core or secondary burnout symptoms at T1.*

|  | Core burnout symptoms (T2) | | | | | | Secondary burnout symptoms (T2) | | | | | |
| --- | --- | --- | --- | --- | --- | --- | --- | --- | --- | --- | --- | --- |
|  | Model 1 | | | Model 2 | | | Model 1 | | | Model 2 | | |
|  | *b* | *SE* | *p* | *b* | *SE* | *p* | *b* | SE | *p* | *b* | SE | *p* |
| Constant | 3.71 | .30 | <.001 | .97 | .23 | <.001 | 3.37 | .31 | <.001 | .93 | .26 | <.001 |
| Gender | -.13 | .09 | .13 | -.10 | .06 | .09 | .05 | .09 | .58 | -.01 | .07 | .84 |
| Age | -.30 | .04 | <.001 | -.06 | .03 | .03 | -.26 | .04 | <.001 | -.05 | .03 | .13 |
| Educational level | -.05 | .08 | .51 | -.01 | .05 | .89 | -.11 | .08 | .18 | -.03 | .06 | .59 |
| Burnout symptoms T1 |  |  |  | .78 | .04 | <.001 |  |  |  | .72 | .04 | <.001 |
| Transportation physical activity T1 |  |  |  | -.01 | .01 | .32 |  |  |  | .01 | .01 | .69 |
| Household physical activity T1 |  |  |  | <.01 | .01 | .84 |  |  |  | .01 | .02 | .73 |
| Recreation physical activity T1 |  |  |  | <.01 | .01 | .60 |  |  |  | -.01 | .01 | .60 |
| *F* | 21.38 |  |  | 95.86 |  |  | 12.63 |  |  | 61.73 |  |  |
| *R^2^* | .15 |  |  | .66 |  |  | .10 |  |  | .56 |  |  |
| Adjusted *R^2^* | .15 |  |  | .65 |  |  | .09 |  |  | .55 |  |  |
| Δ*R^2^* |  |  |  | .51 |  |  |  |  |  | .46 |  |  |

**Testing Hypothesis 3 Cross-sectionally and longitudinally**

Table 3A. *Cross-sectional regression models predicting core and secondary burnout symptoms at T1 from the interaction between physical job demands and different types of off-job physical activity at T1.*

|  | Core burnout symptoms (T1) | | | | | | | | | Secondary burnout symptoms (T1) | | | | | | | | |
| --- | --- | --- | --- | --- | --- | --- | --- | --- | --- | --- | --- | --- | --- | --- | --- | --- | --- | --- |
|  | Model 1 | | | Model 2 | | | Model 3 | | | Model 1 | | | Model 2 | | | Model 3 | | |
|  | *b* | *SE* | *p* | *b* | *SE* | *p* | *b* | *SE* | *p* | *b* | SE | *p* | *b* | SE | *p* | *b* | SE | *p* |
| Constant | 3.56 | .30 | <.001 | 2.70 | .31 | <.001 | 3.20 | .42 | <.001 | 3.34 | .31 | <.001 | 2.40 | .31 | <.001 | 2.96 | .43 | <.001 |
| Gender | -.06 | .09 | .50 | -.05 | .08 | .58 | -.04 | .08 | .59 | .09 | .09 | .32 | .10 | .08 | .25 | .10 | .08 | .25 |
| Age | -.31 | .04 | <.001 | -.26 | .04 | <.001 | -.25 | .04 | <.001 | -.29 | .04 | <.001 | -.23 | .04 | <.001 | -.23 | .04 | <.001 |
| Educational level | -.04 | .08 | .58 | -.02 | .08 | .81 | -.03 | .07 | .68 | -.11 | .08 | .19 | -.06 | .08 | .42 | -.07 | .08 | .36 |
| Transportation physical activity T1 |  |  |  | .03 | .02 | .09 | -.01 | .04 | .90 |  |  |  | .01 | .02 | .42 | -.02 | .04 | .67 |
| Household physical activity T1 |  |  |  | <.01 | .02 | .87 | .02 | .05 | .61 |  |  |  | <.01 | .02 | .79 | <.01 | .05 | .96 |
| Recreation physical activity T1 |  |  |  | -.03 | .01 | .12 | -.12 | .03 | <.001 |  |  |  | -.03 | .02 | .09 | -.12 | .03 | .001 |
| Physical job demands T1 |  |  |  | .32 | .05 | <.001 | .09 | .14 | .53 |  |  |  | .37 | .05 | <.001 | .12 | .15 | .43 |
| Transportation physical activity T1 x Physical job demands T1 |  |  |  |  |  |  | .01 | .02 | .43 |  |  |  |  |  |  | .01 | .02 | .50 |
| Household physical activity T1 x Physical job demands T1 |  |  |  |  |  |  | -.02 | .02 | .37 |  |  |  |  |  |  | -.01 | .02 | .67 |
| Recreational physical activity T1 x Physical demands T1 |  |  |  |  |  |  | .05 | .02 | .001 |  |  |  |  |  |  | .05 | .02 | <.01 |
| *F* | 22.03 |  |  | 18.91 |  |  | 14.97 |  |  | 16.56 |  |  | 18.27 |  |  | 14.18 |  |  |
| *R^2^* | .16 |  |  | .28 |  |  | .30 |  |  | .12 |  |  | .27 |  |  | .29 |  |  |
| Adjusted *R^2^* | .15 |  |  | .26 |  |  | .28 |  |  | .12 |  |  | .26 |  |  | .27 |  |  |
| Δ*R^2^* |  |  |  | .12 |  |  | .03 |  |  |  |  |  | .15 |  |  | .02 |  |  |

Table 3B. *Cross-sectional regression models predicting core and secondary burnout symptoms at T2 from the interaction between physical job demands and different types of off-job physical activity at T2.*

|  | Core burnout symptoms (T2) | | | | | | | | | Secondary burnout symptoms (T2) | | | | | | | | |
| --- | --- | --- | --- | --- | --- | --- | --- | --- | --- | --- | --- | --- | --- | --- | --- | --- | --- | --- |
|  | Model 1 | | | Model 2 | | | Model 3 | | | Model 1 | | | Model 2 | | | Model 3 | | |
|  | *b* | *SE* | *p* | *b* | *SE* | *p* | *b* | *SE* | *p* | *b* | SE | *p* | *b* | SE | *p* | *b* | SE | *p* |
| Constant | 3.71 | .30 | <.001 | 2.91 | .32 | <.001 | 2.64 | .40 | <.001 | 3.37 | .31 | <.001 | 2.29 | .33 | <.001 | 1.60 | .41 | <.001 |
| Gender | -.13 | .09 | .13 | -.11 | .09 | .19 | -.09 | .08 | .27 | .05 | .09 | .58 | .09 | .09 | .31 | .11 | .09 | .19 |
| Age | -.30 | .04 | <.001 | -.24 | .04 | <.001 | -.22 | .04 | <.001 | -.26 | .04 | <.001 | -.19 | .04 | <.001 | -.17 | .04 | <.001 |
| Educational level | -.05 | .08 | .51 | -.02 | .08 | .77 | -.02 | .08 | .82 | -.11 | .08 | .18 | -.08 | .08 | .30 | -.08 | .08 | .29 |
| Transportation physical activity T2 |  |  |  | -.01 | .02 | .40 | -.07 | .04 | .09 |  |  |  | .01 | .02 | .75 | -.03 | .04 | .48 |
| Household physical activity T2 |  |  |  | <.01 | .02 | .92 | .14 | .05 | <.01 |  |  |  | .01 | .02 | .69 | .19 | .05 | <.001 |
| Recreation physical activity T2 |  |  |  | <.01 | .02 | .97 | -.09 | .04 | .02 |  |  |  | -.01 | .02 | .54 | -.07 | .04 | .07 |
| Physical job demands T2 |  |  |  | .29 | .05 | <.001 | .39 | .12 | .001 |  |  |  | .35 | .05 | <.001 | .66 | .12 | <.001 |
| Transportation physical activity T2 x Physical job demands T2 |  |  |  |  |  |  | .03 | .02 | .09 |  |  |  |  |  |  | .02 | .02 | .20 |
| Household physical activity T2 x Physical job demands T2 |  |  |  |  |  |  | -.08 | .02 | <.001 |  |  |  |  |  |  | -.10 | .02 | <.001 |
| Recreational physical activity T2 x Physical demands T2 |  |  |  |  |  |  | .05 | .02 | <.01 |  |  |  |  |  |  | .04 | .02 | .06 |
| *F* | 21.38 |  |  | 15.21 |  |  | 12.80 |  |  | 12.63 |  |  | 13.87 |  |  | 11.99 |  |  |
| *R^2^* | .15 |  |  | .24 |  |  | .27 |  |  | .10 |  |  | .22 |  |  | .26 |  |  |
| Adjusted *R^2^* | .15 |  |  | .22 |  |  | .25 |  |  | .09 |  |  | .20 |  |  | .24 |  |  |
| Δ*R^2^* |  |  |  | .08 |  |  | .04 |  |  |  |  |  | .12 |  |  | .04 |  |  |

Table 3C. *Longitudinal regression models predicting core and secondary burnout symptoms at T2 from the interaction between physical job demands and different types of off-job physical activity at T1, controlling for core or secondary burnout symptoms at T1.*

|  | Core burnout symptoms (T2) | | | | | | | | | Secondary burnout symptoms (T2) | | | | | | | | |
| --- | --- | --- | --- | --- | --- | --- | --- | --- | --- | --- | --- | --- | --- | --- | --- | --- | --- | --- |
|  | Model 1 | | | Model 2 | | | Model 3 | | | Model 1 | | | Model 2 | | | Model 3 | | |
|  | *b* | *SE* | *p* | *b* | *SE* | *p* | *b* | *SE* | *p* | *b* | SE | *p* | *b* | SE | *p* | *b* | SE | *p* |
| Constant | 3.71 | .30 | <.001 | .93 | .24 | <.001 | 1.30 | .32 | <.001 | 3.37 | .31 | <.001 | .81 | .26 | <.01 | .89 | .36 | .01 |
| Gender | -.13 | .09 | .13 | -.08 | .06 | .17 | -.08 | .06 | .14 | .05 | .09 | .58 | <.01 | .07 | .99 | <.01 | .07 | .99 |
| Age | -.30 | .04 | <.001 | -.05 | .03 | .07 | -.05 | .03 | .07 | -.26 | .04 | <.001 | -.05 | .03 | .15 | -.05 | .03 | .15 |
| Educational level | -.05 | .08 | .51 | -.03 | .05 | .63 | -.02 | .05 | .70 | -.11 | .08 | .18 | -.06 | .06 | .31 | -.06 | .06 | .30 |
| Burnout symptoms T1 |  |  |  | .78 | .04 | <.001 | .77 | .04 | <.001 |  |  |  | .71 | .04 | <.001 | .71 | .04 | <.001 |
| Transportation physical activity T1 |  |  |  | .01 | .01 | .58 | .01 | .03 | .71 |  |  |  | .02 | .01 | .12 | .01 | .03 | .63 |
| Household physical activity T1 |  |  |  | -.01 | .01 | .28 | -.06 | .03 | .08 |  |  |  | .01 | .02 | .67 | .01 | .04 | .80 |
| Recreation physical activity T1 |  |  |  | .01 | .01 | .41 | -.01 | .02 | .58 |  |  |  | .01 | .011 | .49 | -.01 | .03 | .84 |
| Physical job demands T1 |  |  |  | <.01 | .03 | .94 | -.17 | .10 | .10 |  |  |  | .03 | .04 | .51 | -.01 | .12 | .96 |
| Transportation physical activity T1 x Physical job demands T1 |  |  |  |  |  |  | <.01 | .01 | .76 |  |  |  |  |  |  | <.01 | .01 | .86 |
| Household physical activity T1 x Physical job demands T1 |  |  |  |  |  |  | .02 | .02 | .16 |  |  |  |  |  |  | <.01 | .02 | .88 |
| Recreational physical activity T1 x Physical demands T1 |  |  |  |  |  |  | .01 | .01 | .37 |  |  |  |  |  |  | .01 | .01 | .60 |
| *F* | 21.38 |  |  | 83.55 |  |  | 61.29 |  |  | 12.63 |  |  | 55.24 |  |  | 39.91 |  |  |
| *R^2^* | .15 |  |  | .66 |  |  | .66 |  |  | .10 |  |  | .56 |  |  | .56 |  |  |
| Adjusted *R^2^* | .15 |  |  | .65 |  |  | .65 |  |  | .09 |  |  | .55 |  |  | .55 |  |  |
| Δ*R^2^* |  |  |  | .50 |  |  | <.01 |  |  |  |  |  | .46 |  |  | <.01 |  |  |
